# Comprehensive analysis based on the disulfidptosis-related genes identifies hub genes and immune infiltration for pancreatic adenocarcinoma

**DOI:** 10.1515/med-2024-0906

**Published:** 2024-03-04

**Authors:** Yu Li, Miao-xuan Chen, Hai-tao Li, Xiao-ming Cai, Bo Chen, Ze-feng Xie

**Affiliations:** The First Affiliated Hospital of Shantou University Medical College, Shantou, Guangdong, China

**Keywords:** disulfidptosis, pancreatic adenocarcinoma, machine learning, prognostic model, immune infiltration

## Abstract

Pancreatic adenocarcinoma (PAAD) is a prevalent and aggressive malignancy in the digestive tract, requiring accurate prediction and effective treatment strategies. Recently, the discovery of disulfidptosis, a novel form of programmed cell death characterized by abnormal disulfide accumulation, has sparked interest in its role in PAAD. In this study, we aimed to investigate the involvement of disulfidptosis-related genes (DRGs) in PAAD. Using publicly available databases, we conducted a comprehensive analysis exploring the complex relationships between DRGs and important aspects of PAAD, including gene expression, immune response, mutation, drug sensitivity, and functional enrichment. Notably, we observed significant heterogeneity among different disulfidptosis subclusters and identified specific differentially expressed genes in PAAD. Through machine learning techniques, we identified SLC7A11, S100A4, DIAPH3, PRDX1, and SLC7A7 as the most relevant hub genes. We further validated their significance in PAAD by considering their expression patterns, prognostic value, diagnostic potential, diagnostic model, and immune infiltration. This study presents exciting opportunities and challenges in unraveling the underlying mechanisms of PAAD prognosis. It also establishes a foundation for personalized cancer care and the development of innovative immunotherapeutic strategies. By shedding light on the role of DRGs, particularly hub genes, we enhance our understanding and management of PAAD.

## 1 Introduction

Pancreatic adenocarcinoma (PAAD) is a highly malignant cancer of the abdomen with rapid spread and poor prognosis [1,2]. By 2030, pancreatic ductal adenocarcinoma is projected to become the second leading cause of cancer-related deaths in the United States. PAAD is a highly lethal disease with poor prognosis, posing significant challenges for diagnosis and treatment [3]. PAAD has a dismal prognosis with a median survival of <6 months and a 5-year survival rate of approximately 8%, largely due to inadequate early detection methods [4]. Hence, there is a critical need for the development of efficient and sensitive diagnostic markers to predict prognosis and halt the progression of PAAD. Emerging research suggests the existence of a novel mode of cell death known as disulfidptosis. Unlike the well-known programmed cell death mechanisms such as apoptosis, ferroptosis, necroptosis, and cuproptosis, disulfidptosis operates independently. It is characterized by a rapid cell death process induced by disulfide stress, which arises from the buildup of excessive intracellular cystine. This novel mode of cell death highlights the complexity of cellular responses to various stressors and presents potential avenues for further exploration in the field of cell biology and disease mechanisms [5]. Previous research has revealed that in cells overexpressing SLC7A11, a transporter involved in cystine uptake, there is a significant depletion of NADPH and abnormal accumulation of disulfides, specifically cystine, under glucose starvation conditions. This leads to the induction of disulfide stress and subsequent rapid cell death [6]. These findings highlight the intricate interplay between cellular metabolism, redox balance, and cell survival, shedding light on the potential role of SLC7A11 and disulfide stress in cellular responses to nutrient deprivation and their implications in various diseases. Both the endoplasmic reticulum in eukaryotic cells and the periplasmic space in prokaryotic cells play crucial roles in the formation and transfer of protein disulfide bonds. This process is catalytic and involves the participation of numerous proteins and small molecules to facilitate the formation of structural disulfide bonds [7]. Disulfide bonds are prevalent in chemistry, biology, and materials science. Polymer nanomaterials incorporating disulfide bonds possess excellent properties and hold great potential as carriers for drug and gene delivery [8]. With the identification of disulfide bond formation in cancer-related proteins, it is crucial to explore targeting these allosteric bonds for innovative therapies. By focusing on disrupting or modulating the formation of disulfide bonds, new treatment strategies can be developed to interfere with key pathways in cancer progression, offering potential breakthroughs in cancer therapy [9]. Currently, disulfidptosis has been shown to be associated with tumorigenicity in a variety of tumors, including bladder cancer [10], hepatocellular carcinoma [11], and lung adenocarcinoma [12]. Nevertheless, the impact of disulfidptosis on the prognosis and immune infiltration of PAAD is not yet clear and requires further investigation to gain a deeper understanding. Additional research is needed to explore the potential significance of disulfidptosis in PAAD prognosis and its association with immune infiltration.

To investigate potential pathogenic mechanisms, we conducted an analysis of differentially expressed genes (DEGs) between PAAD samples and normal tissue using publicly available databases such as the Cancer Genome Atlas (TCGA) and Gene Expression Omnibus (GEO). We focused on exploring the expression, immunity, mutation, and drug sensitivity of these DEGs in PAAD. Subsequently, we constructed sub-clusters of disulfidptosis-related genes (DRGs) based on clinical features and gene expression patterns, aiming to uncover associated mechanisms. We identified overlapping genes between the differentially expressed DEGs and DRGs. Furthermore, machine learning algorithms were applied to identify key DRGs such as S100A4, SLC7A11, PRDX1, SLC7A7, and DIAPH3. Finally, we identified the strongest trait gene and investigated its relationship with diagnosis, prognosis, and immune infiltration in PAAD. This comprehensive approach offers a new perspective to gain a deeper understanding of the underlying molecular mechanisms driving PAAD pathogenesis.

## 2 Materials and methods

### 2.1 Identification of DRGs

In our study, we initially identified 18 DRGs based on previous literature [5,11]. Based on the findings from this study, we focused on selecting DRGs that were repeatedly and distinctly mentioned in relation to disulfidptosis, a cellular process of interest. Each of these genes encodes specific proteins that play significant roles in biochemical pathways and cellular structures involved in disulfidptosis. For example, G6PD and PGD code for enzymes in the pentose phosphate pathway, while FLNA, MYH9, MYH10, ACTB, and TLN1 are involved in regulating actin cytoskeleton dynamics. SLC7A11, which codes for a cystine transporter, was particularly emphasized due to its central role in disulfidptosis. Furthermore, the selection of genes took into consideration their interactions with the WAVE regulatory complex (WRC), a key player in actin polymerization and lamellipodia formation, both of which are processes associated with disulfidptosis. Genes such as NCKAP1, NCKAP1L, WASF2, CYFIP1, ABI2, BRK1, and RAC1 were chosen for their interactions with the WRC. Additionally, SLC3A2 and RPN1 were noted for their specific roles in modulating disulfidptosis. PRDX1, which encodes peroxiredoxin-1, a known disulfide-bonded protein with established roles in redox maintenance, was also included in the selection. Overall, this process of gene selection provided an overview of the gene landscape associated with disulfidptosis, shedding light on the various cellular components and processes involved. In total, we identified 18 candidate genes associated with disulfidptosis.

To further explore their functional associations, we utilized the GeneMANIA Prediction Server, a tool for gene prioritization and function prediction through biological network integration [13]. By accessing the GeneMANIA website (http://www.genemania.org), we identified an additional set of 40 DRGs, which were functionally similar to the initial set of genes. This expanded set of DRGs provides a comprehensive view of the potential functional relationships and pathways associated with the initial set of genes.

### 2.2 PAAD datasets

PAAD is a type of cancer that originates in the cells lining the pancreas. It is the most common form of pancreatic cancer and is known for its aggressive nature and poor prognosis. PAAD typically develops in the exocrine cells of the pancreas, which are responsible for producing enzymes involved in digestion [14]. UCSC XENA (https://xenabrowser.net/datapages/) RNAseq data from TCGA and genotype-tissue expression (GTEx) are processed uniformly by the Toil process [15]. Extracted PAAD data corresponding to TCGA (http://cancergenome.nih.gov) and corresponding normal tissue data in GTEx (https://www.gtexportal.org/home/-index.html) with de-batch effect. We obtained RNA-sequencing (RNA-seq) data and clinical data from 179 PAAD tumor samples and four paraneoplastic tissues from TCGA. We also extracted the relevant normal data (*n* = 167) for PAAD from the GTEx database. Additionally, they included the GSE15471 [16] dataset from GEO (https://www.ncbi.nlm.nih.gov/geo/), which consists of 39 PAAD cancer samples and 39 paired normal samples. Another dataset used was GSE16515 [17], which contains 36 PAAD cancer samples and 16 paired normal samples. These datasets were utilized for various analyses, such as examining the expression levels of DRGs, performing differential gene analysis, employing machine learning techniques, investigating prognosis, and assessing immune infiltration, among other analyses. The data obtained from these sources were in TPM format, and the researchers performed a log2(*x* + 1) transformation on each expression value. All data analysis was conducted using R (version 4.2.1) and relevant bioinformatics analysis websites.

### 2.3 Gene set and DEGs functional enrichment analysis

For gene set functional enrichment, we utilized the KEGG rest API (https://www.kegg.jp/kegg/rest/keggapi.html) to retrieve the most recent gene annotations from the KEGG pathway database as the background set. We then mapped our genes of interest to this background set and conducted the enrichment analysis using the R package ClusterProfiler (version 3.14.3) [18]. This analysis allowed us to obtain the results of gene set enrichment, identifying pathways that were significantly enriched with our genes of interest. Similarly, for gene ontology (GO) enrichment analysis, we utilized the GO annotations of genes from the org.Hs.eg.db R package (version 3.1.0) as the background set. We mapped our genes to this background set and performed enrichment analysis using the clusterProfiler R package (version 3.14.3). This analysis helped to identify significantly enriched GO terms associated with our genes of interest. We considered enrichment results with *p* < 0.05 and false discovery rate (FDR) <0.1 as statistically significant.

To analyze the functional enrichment of DEGs, we utilized KEGG enrichment analysis to gain insights into gene function and high-level genomic functional information. Additionally, we employed GO annotations, which provide comprehensive gene function annotations, including molecular function (MF), biological process, and cellular component categories. To investigate the oncogenic and immune infiltration aspects of the target genes, we performed KEGG pathway enrichment analysis for the differentially up-regulated and down-regulated genes. This analysis allowed us to identify enriched pathways associated with these genes using the ClusterProfiler R package (version 3.18.0). Additionally, we conducted GO term enrichment analysis for the differentially up-regulated and down-regulated genes, providing insights into their functional annotations. In both KEGG and GO enrichment analyses, we considered enrichment results with *p* < 0.05 as statistically significant, indicating a potential association between the DEGs and specific pathways or functional categories.

### 2.4 Gene set cancer analysis (GSCA)

GSCA is a comprehensive platform that integrates various analyses for genomic, pharmacogenomic, and immunogenomic cancer research [19]. This enhanced version of GSCA offers a wide range of services to investigate gene set genomics, including gene expression analysis, single nucleotide variation (SNV) analysis, copy number variation (CNV) analysis, methylation analysis, and immunogenomic analysis involving 24 immune cell types.

### 2.5 Sub-cluster analysis

We conducted consistency analysis using the ConsensusClusterPlus R package (version 1.54 4.0) [20] to assess the stability and robustness of clustering results. The analysis was performed with a maximum number of clusters set to 6, and 80% of the total samples were randomly selected 100 times. The clustering algorithm used was hierarchical clustering (clusterAlg = “hc”) with inner linkage method set to “ward.D2”.

To visualize the clustering results, we generated cluster heatmaps using the pheatmap R package (version 1.0.12). In the gene expression heatmaps, we included only genes with a standard deviation greater than 0.1, ensuring that genes with sufficient variability across the samples were retained for visualization and analysis. The heatmaps provided a visual representation of the clustering patterns and gene expression profiles, aiding in the identification of potential clusters and patterns in the data.

### 2.6 Differential genetic screening

Limma (linear models for microarray data) is a differential expression screening method that utilizes a generalized linear model [21]. In the TCGA database, DEGs were screened in subgroup C1 and C2 data sets. Differentially expressed mRNA was studied using the limma package (version 3.40.6). Threshold for differential mRNA expression between two clusters was set at “Adjusted *p* < 0.05 and |log2FC| > 1.5.” In GSE15471 and GSE16515, *p* < 0.05 and |log2FC| > 1 was selected as the cut-off standard. Specifically, GSE15471 and GSE16515 were downloaded from the GEO database using GEOquery [2.64.2] [22]. Missing values in the dataset were handled using the impute package, which filled in the missing values and performed data renormalization using limma’s normalizeBetweenArrays function. The limma package was then utilized to conduct a differential analysis between the two groups in the dataset. The results of this analysis were visualized through volcano plots, showcasing the fold change and statistical significance of gene expression differences. Additionally, heatmaps were generated to visualize the expression patterns of molecules that exhibited significant expression changes [23].

### 2.7 Machine learning

In order to identify trait genes, two machine learning approaches were employed to screen for DRGs. The first method used was the least absolute shrinkage and selection operator (LASSO), which is a regression technique that incorporates regularization to enhance prediction accuracy and model interpretability by selecting relevant variables [24]. The second approach utilized Random Forest, a learning algorithm that constructs numerous decision trees and provides class predictions based on the collective output of these trees. Random Forest is known for its high accuracy, sensitivity, and specificity [25]. Additionally, survival analysis was conducted using the log-rank test to compare survival differences between two groups, while time receiver operating characteristic (ROC) analysis was performed to assess the accuracy of the predictive model.

### 2.8 Statistical analysis

The R package (version 4.2.1) was used to perform all statistical tests, while the ggplot2 package (version 3.3.6) was employed for visualizations. The Wilcoxon rank sum test statistic from the stats package (version 4.2.1) was utilized to analyze differences in the expression of DRGs in PAAD. The igraph package (version 1.3.4) and ggraph package (version 2.1.0) were employed to visualize the expression correlation network of the DRGs. Using the xCell package (version 1.1.0), the integrated level of 64 cell types, including 14 stromal cell types, was estimated. LASSO regression and Random Forest analyses were carried out using the “glmnet” package [26] and the “randomForest” package [27], respectively. Kaplan–Meier survival analyses were performed using the “survival R” and “survminer R” packages (version 3.3.1). ROC analysis was conducted using the qROC package (version 1.18.0). The construction and visualization of Nomogram models were achieved using the rms package (version 6.4.0). Spearman correlation analysis was used to understand the relationship between the expression levels of hub genes and immune infiltration. The immune scores were calculated using the immune infiltration algorithm (ssGSVA) from the GSVA package (version 1.46.0) [28]. The Wilcoxon rank-sum test was employed to compare differences between groups, with statistical significance defined as *p* < 0.05 (ns, *p* ≥ 0.05; ∗*p* < 0.05; ∗∗*p* < 0.01; ∗∗∗*p* < 0.001; ****p < 0.0001).

## 3 Results

### 3.1 Rational definition of the DRGs in PAAD

**Figure 1:**
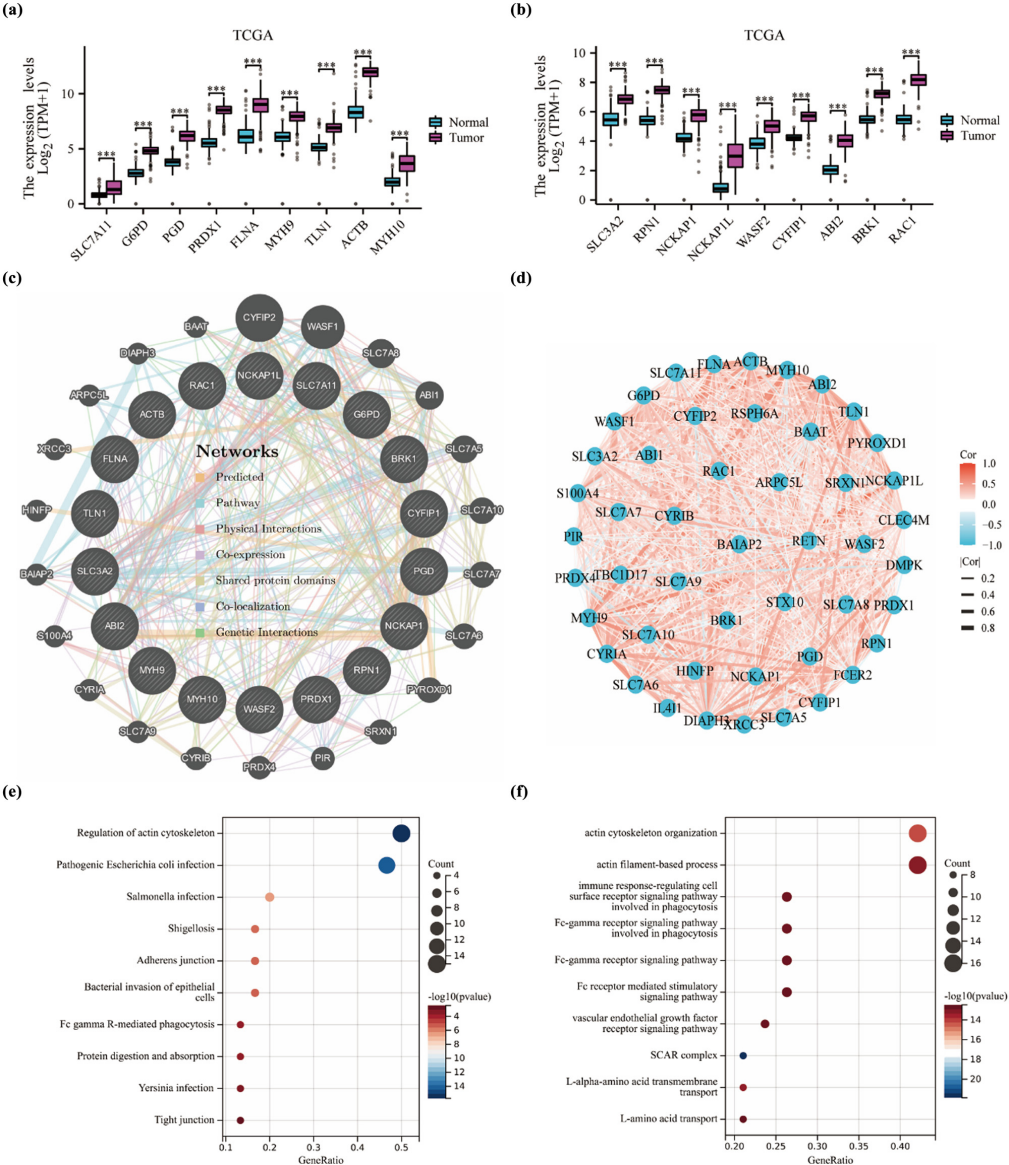
Defining DRGs in PAAD appropriately. (a and b) Expression distributions of 18 DRGs between cancer and normal tissues in TCGA and GTEx dataset. (c) Establish 40 DRGs with similar functions using GeneMANIA. The seven lines are colored to indicate the type of gene interaction. (d) Network diagram showing the correlation of the expression of DRGs in the TCGA dataset. (e) KEGG and (f) GO concentrated air bubble diagram. ***p < 0.001.

In previous studies, a set of 18 genes (SLC7A11, G6PD, PGD, PRDX1, FLNA, MYH9, TLN1, ACTB, MYH10, SLC3A2, RPN1, NCKAP1, NCKAP1L, WASF2, CYFIP1, ABI2, BAK1, and RAC1) have been identified as being associated with disulfidptosis. [11]. To validate the expression patterns of these aforementioned genes in PAAD, we obtained expression data from the TCGA and GTEx databases for both cancerous and normal tissues. Analysis of the data revealed significant differences in the expression levels of the DRGs between PAAD and normal tissues. The expression of all relevant genes was up-regulated and significant in the tumors (Figure 1a and b). GeneMANIA was employed to predict functionally similar genes among the hub genes. We obtained 22 similar gene hub genes, comprising CYFIP2, WASF1, SLC7A8, ABI1, SLC7A5, SLC7A10, SLC7A7, SLC7A6, PYROXD1, SRXN1, PIR, PRDX4, CYRIB, SLC7A9, CYRIA, S100A4, BAIAP2, HINFP, XRCC3, ARPC5L, DIAPH3 and BAAT. In the analysis, the hub gene was positioned in the inner circle, while the predicted genes were situated in the outer circle. The relationships between the genes were determined based on seven specific types, including Predicted, Pathway, Physical Interactions, Co-expression, Shared protein domains, Co-localization, and Genetic Interactions (Figure 1c). Network diagram based on correlation analysis of the TCGA dataset, analyzing that the expression of most of the genes of interest are positively correlated with each other (Figure 1d). Enrichment analysis of DRGs in the KEGG dataset identified some pathways such as PAAD. Further enrichment analysis of these genes on the GO dataset indicated that certain related actin nucleation items, such as actin cytoskeleton organization, actin filament-based process and immune response-regulating cell surface receptor signaling pathway involved in phagocytosis and so on (Figure 1f). These preliminary exploratory analyses serve as a supportive foundation for characterizing DRGs and elucidating their associated pathway mechanisms. Consequently, we have identified 40 DRGs that have been selected as promising candidate genes for further in-depth investigation.

### 3.2 Investigating the expression, immune response, mutations, and drug sensitivity of 29 DRGs in PAAD

**Figure 2:**
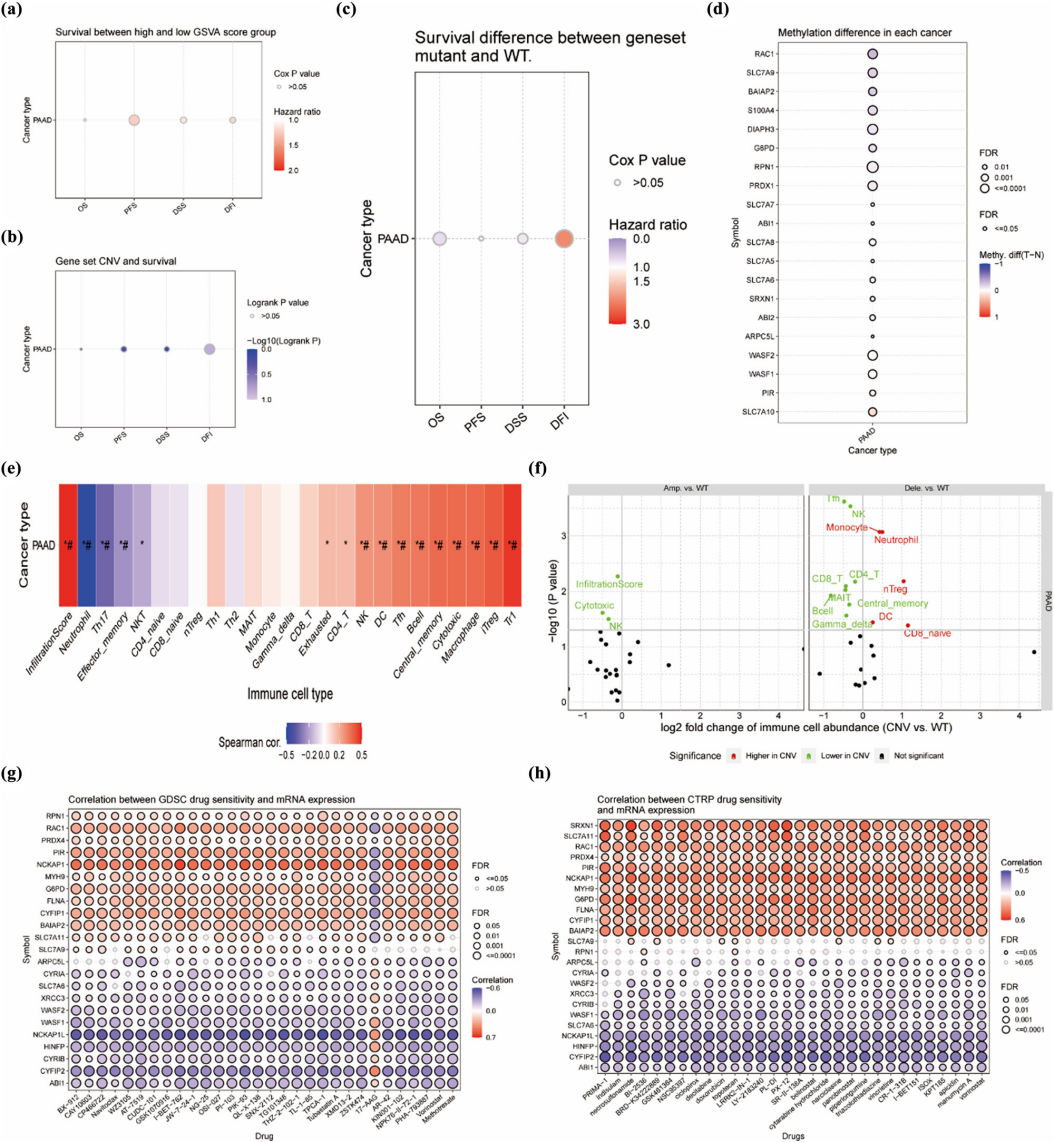
Thorough analysis of 40 DRGs in PAAD. (a) Survival difference between high and low GSVA score groups in PAAD. (b) Summarizes the profile of survival between gene set CNV groups in PAAD. (c) Survival discrepancy observed between gene set mutant (deleterious) and WT in PAAD. When hazard ratio is less than 1 and close to 0, the blue color is darker; when hazard ratio is greater than 1, the red color is darker and the value increases. (d) Methylation difference between tumor and normal samples of DRGs in PAAD. (e) Correlation of DRGs expression with different immune cell types in PAAD. (f) Differences in immune infiltration between genomic CNV groups are summarized. (g) Correlation of DRGs with GDSC drug sensitivity (top 30) in pan-cancer patients. (h) Correlation of DRGs with CTRP drug sensitivity (top 30) in pan-cancer patients. DFI: disease-free interval; DSS: disease specific survival; OS: overall survival; PFI: progression-free survival; GSVA score: gene set expression score; CNV: copy number variation; SNV: single nucleotide variation; Amp: amplification; Dele: deleton; WT: wild type; GDSC: genomics of drug sensitivity in cancer; CTRP: The Cancer Therapeutics Response Portal. Cox *p* value > 0.05 was statistically significant and was statistically valuable with the larger the dots. *p < 0.05, #FDR < 0.05.

To obtain a comprehensive understanding of the role and significance of DRGs in cancer diagnosis, we employed GSCA to perform a correlated analysis of four modules: expression, immunity, mutations, and drug sensitivity. By employing this approach, we obtained valuable insights into the dynamic interplay between DRGs and critical facets of cancer biology. In the expression module, we found statistically significant OS, PFS, DSS, and DFI between the GSVA score groups for PAAD (Figure 2a). In the mutation module, we found statistically significant correlations between CNV of DRGs and survival prognosis (OS, PFS, DSS, and DFI) (Figure 2b). Furthermore, the presence or absence of mutations in DRGs also had an impact on survival outcomes in PAAD patients and was most strongly associated with DFI (Figure 2c). In addition, DRGs are strongly linked to methylation. Among them, methylation was most strongly positively correlated with SLC7A10 than negatively correlated with RAC1 (Figure 2d). In the immunity module, DRGs positively correlate with overall trend of immune infiltration in PAAD. DRGs were most meaningfully and positively correlated with Tr1 (*p* < 0.05, #FDR < 0.05) and most negatively correlated with neutrophil (*p* < 0.05, #FDR < 0.05) (Figure 2e). Interestingly, Figure 2f summarizes the difference of immune infiltration between higher and lower CNV groups. In amplification CNV, InfiltrationScore, cytotoxic, and NK are meaningfully low expressed in CNV. In deleton CNV, CD8 native, DC, nTreg monocyte, and neutrophil are meaningfully highly expressed in CNV, as opposed to CD4 T, CD8 T, NK, B cell, central memory, and so on. Furthermore, the correlation analysis of gene expression with GDSC drug sensitivity (top 30) (Figure 2g) and CTRP drug sensitivity (top 30) (Figure 2h) across various cancer types demonstrated consistent and reliable results. In conclusion, the comprehensive analysis of multiple modules described above revealed robust associations between DRGs and crucial aspects such as gene expression, immune infiltration, mutations, and drug sensitivity in PAAD. This analysis deepened our understanding of the intricate involvement of DRGs in these key biological processes, providing valuable insights into their significance in cancer. Through GSCA, researchers can leverage clinical information and small molecule drug data to identify potential biomarkers and valuable small drugs. This information can be utilized to guide further clinical trials and enhance personalized medicine approaches in cancer research. Overall, GSCA provides a comprehensive suite of tools and services that enable researchers to perform integrated genomic, pharmacogenomic, and immunogenomic analyses, facilitating a better understanding of cancer biology and aiding in the identification of potential therapeutic targets and treatment strategies.

### 3.3 Identification and analysis of two sub-clusters of disulfidptosis and differential gene expression in sub-clusters

**Figure 3:**
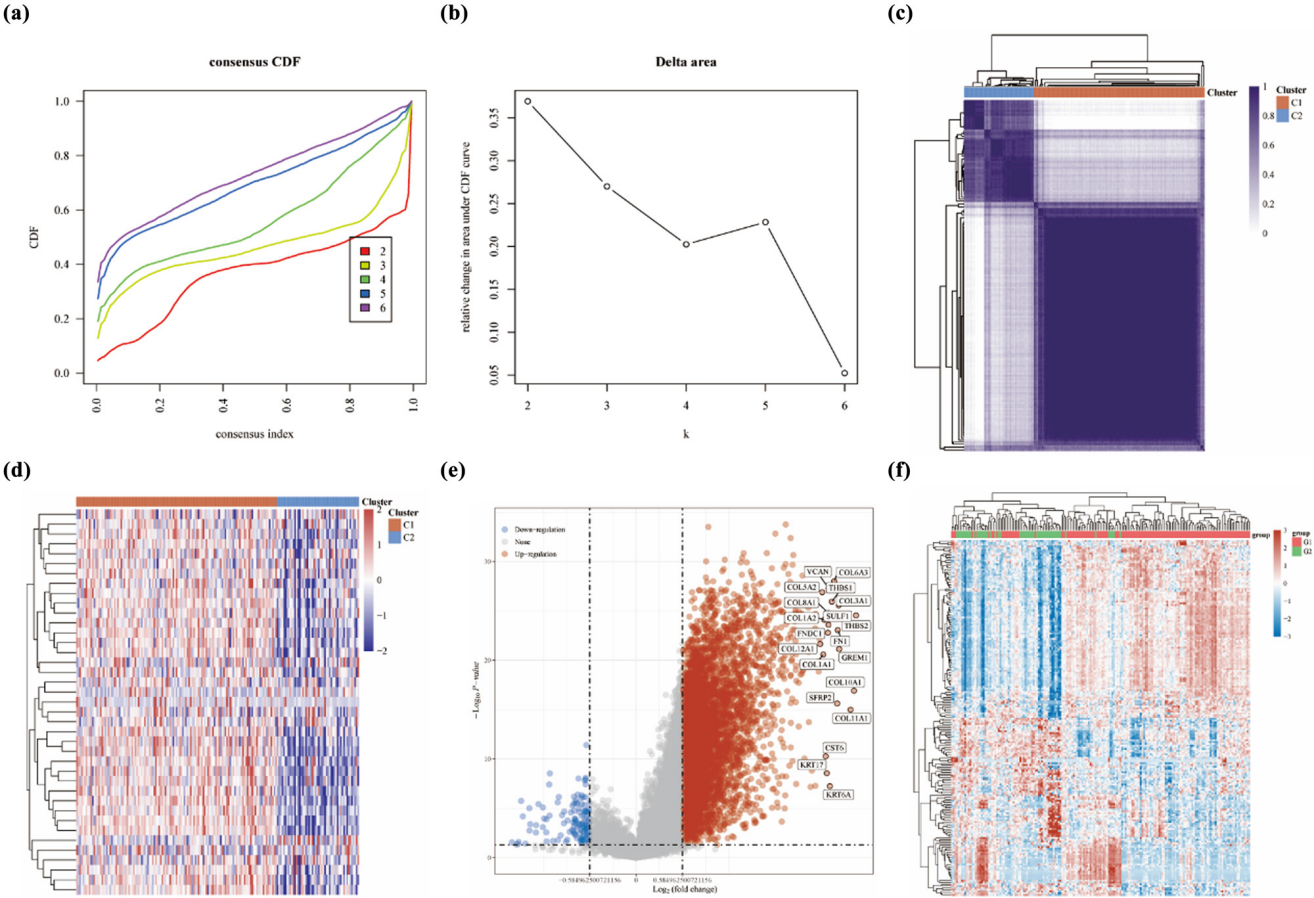
Two distinct sub-clusters of disulfidptosis were identified. (a and b) CDF curves were generated for *K* values ranging from 2 to 6. We assessed the relative change in the area under the CDF curve for each *K* value. (c) Unsupervised clustering analysis with *k* = 2 was performed to identify appropriate clusters. (d) A heatmap was generated to visualize the expression relationship of DRGs within different subgroups. (e) Volcano map showing grouped differential gene analysis. (f) Heatmap showing the most significant differences in gene expression.

**Figure 4:**
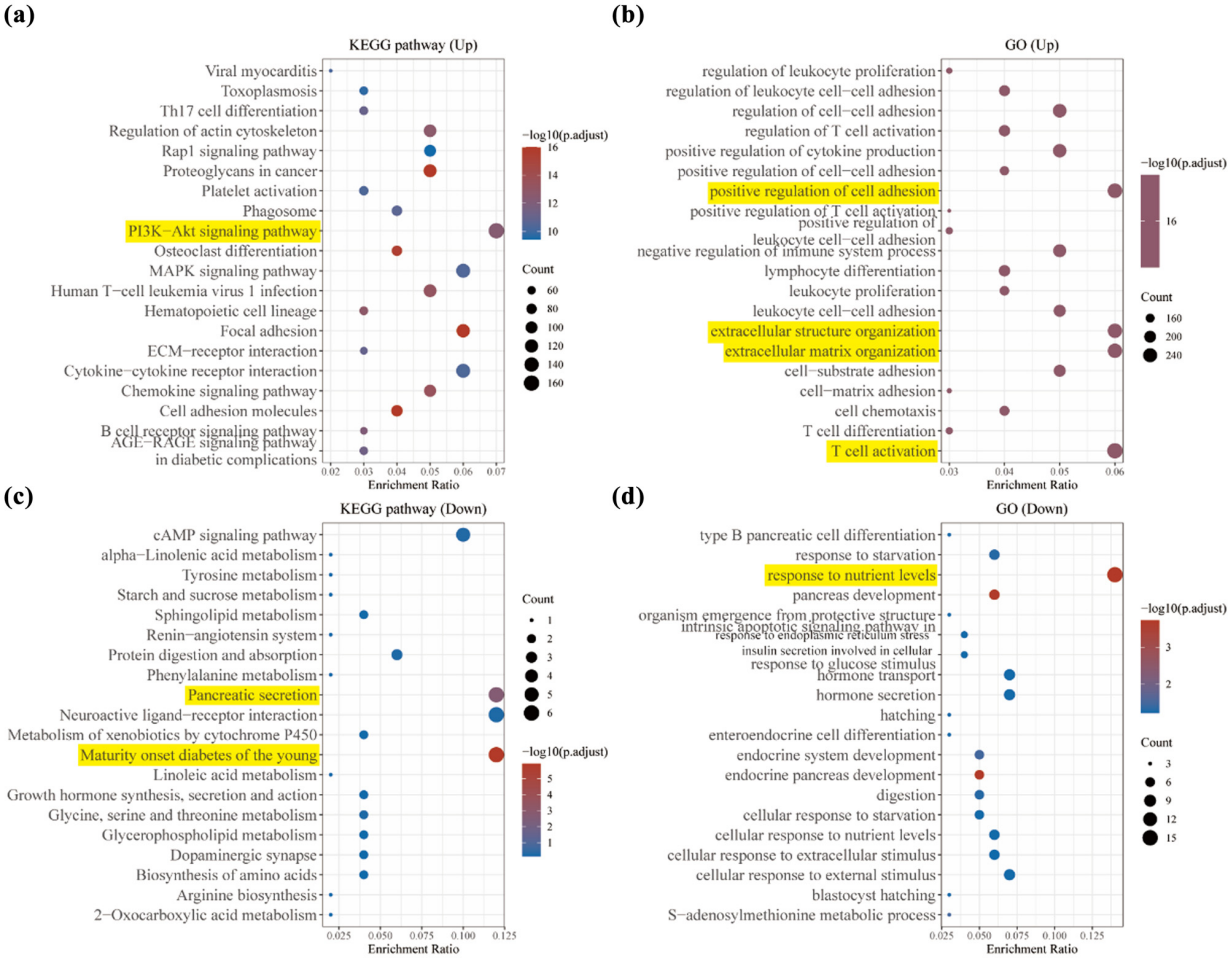
Differential genes in subgroups enriched in KEGG and GO pathway. (a) Up-regulation of genes in KEGG enrichment-associated pathways. (b) Up-regulation of genes in GO enrichment-associated pathways. (c) Down-regulation of genes in KEGG enrichment-associated pathways. (d) Down-regulation of genes in GO enrichment-associated pathways. In the enrichment analysis, pathways with *p*-values less than 0.05 were considered significant, indicated by an enrichment score with a −log10(*P*) value greater than 1.3, suggesting their relevance and significance. Yellow underlining emphasizes the representative enrichment of the pathway.

We conducted unsupervised consensus clustering on a cohort of 179 patients with PAAD using data obtained from TCGA databases. Our objective was to maximize the area under the cumulative distribution function (CDF) curve while considering the decreasing trend of CDF Delta. By carefully evaluating these two factors, we determined that the optimal number of clusters (*K*) was 2 (Figure 4a and b). In addition, when *K* = 2, two sub-clusters (Cluster 1 [*n* = 124], Cluster 2 [*n* = 55]) were selected to have relative stability and reliability under the distribution (Figure 4c). In contrast to the C2 sub-group, DRGs were highly expressed in the C1 sub-cluster (Figure 3d). To further explore the differences between the two sub-clusters, Limma analysis of the volcano map (Figure 3e) and heatmap (Figure 3f) was used to demonstrate the differential genes between the two sub-clusters. There were 4,652 up-regulated genes and 126 down-regulated genes between the two sub-clusters (Figure 3e). The heatmap further demonstrates that the differential genes have expression differences between the two sub-clusters (Figure 3f). Enrichment analysis of the up-regulated genes in the KEGG dataset are shown in PI3K-Akt signaling pathway (Figure 4a), compared to the down-regulated genes in maturity onset diabetes of the young (Figure 4c). The further enrichment analysis of the up-regulated genes in the GO dataset are shown in positive regulation of T cell adhesion, extracellular structure organization, extracellular matrix organization, and T cell activation (Figure 4b), compared to the down-regulated genes in response to nutrient levels, which were involved in immune response (Figure 4d). The up-regulated genes in this pathway may suggest dysregulation of PI3K-Akt signaling in the studied PAAD C1 subtype. This pathway is known to play a crucial role in cell growth, survival, and proliferation, and its alteration has been implicated in various cancers, including pancreatic cancer [29]. The dysregulation of this pathway in PAAD could contribute to enhanced cell survival, proliferation, and resistance to apoptosis. This will help to understand that cell death is more linked in the C1 subgroup.

**Figure 5:**
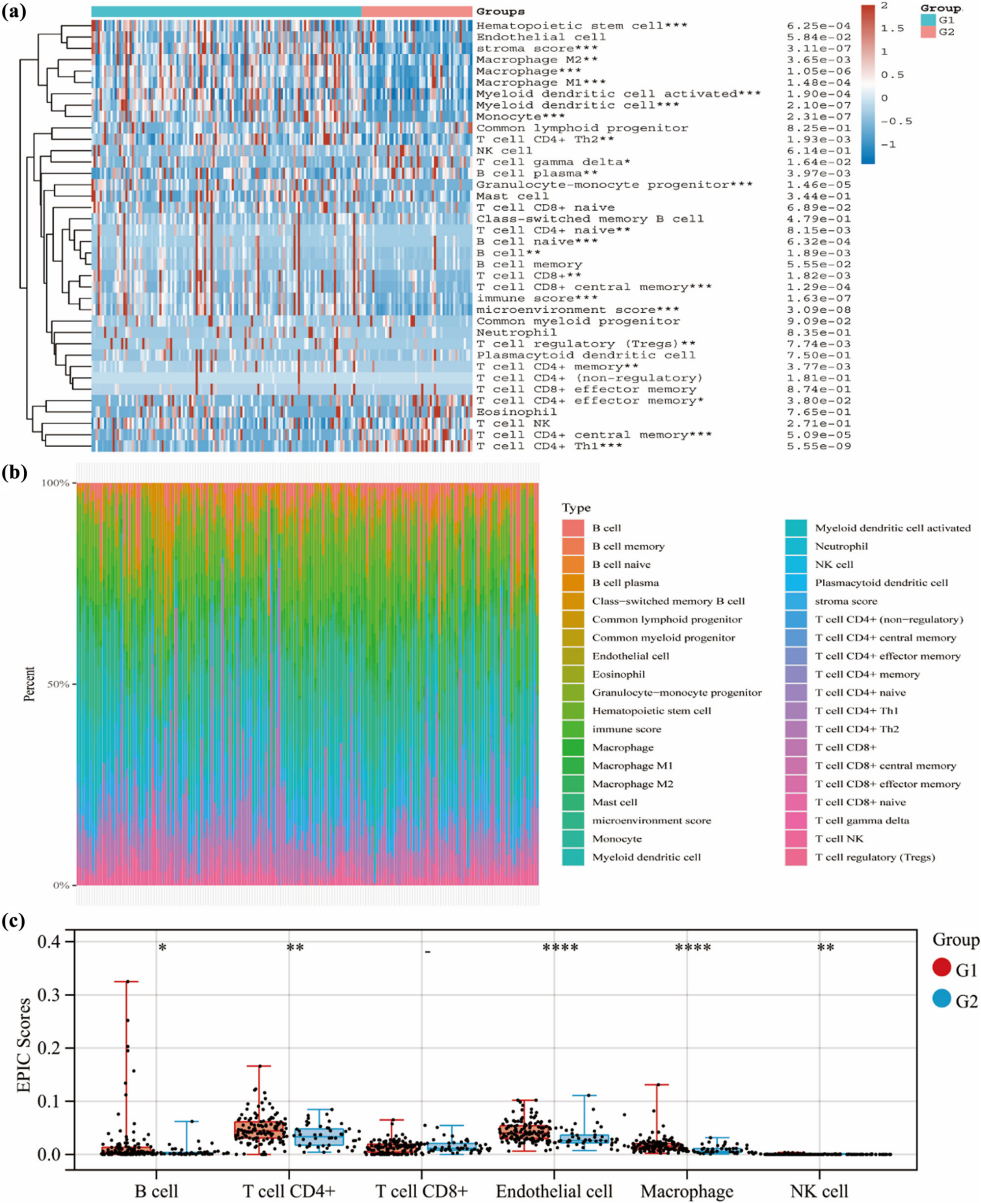
Immune cells infiltration between sub-groups. (a) Immune cells infiltration between different groups by xCell algorithms. (b) The proportion structure of all immune cell types. (c) The expression of seven key immune checkpoint genes in two sub-groups. G1 represents the C1 subgroup and G2 represents the C2 subgroup. *p < 0.05, **p < 0.01, ***p < 0.001, and ****p < 0.0001.

### 3.4 Immune cell composition in PAAD

To gain a deeper understanding of the immune system’s involvement in PAAD, we utilized the xCell algorithm to examine the composition of immune cells. Furthermore, we employed the xCell algorithm to analyze the immunological features of different subgroups within the study (*p* < 0.05) (Figure 5a). In contrast to the C2 subgroup, the C1 subgroup exhibited a significant positive correlation with immune cells. Figure 5b illustrates the proportions of various immune cell types. Additionally, we examined the expression levels of crucial immune checkpoint genes within these subgroups to predict the potential efficacy of immune checkpoint blockade therapy (Figure 5c). The results showed that the expressions of B cell, T cell CD4+, endothelial cell, macrophage, and NC cell were elevated in C1 subgroup (*p* < 0.01) (Figure 5c). These findings highlight the critical role of immunological processes in developing PAAD.

**Figure 6:**
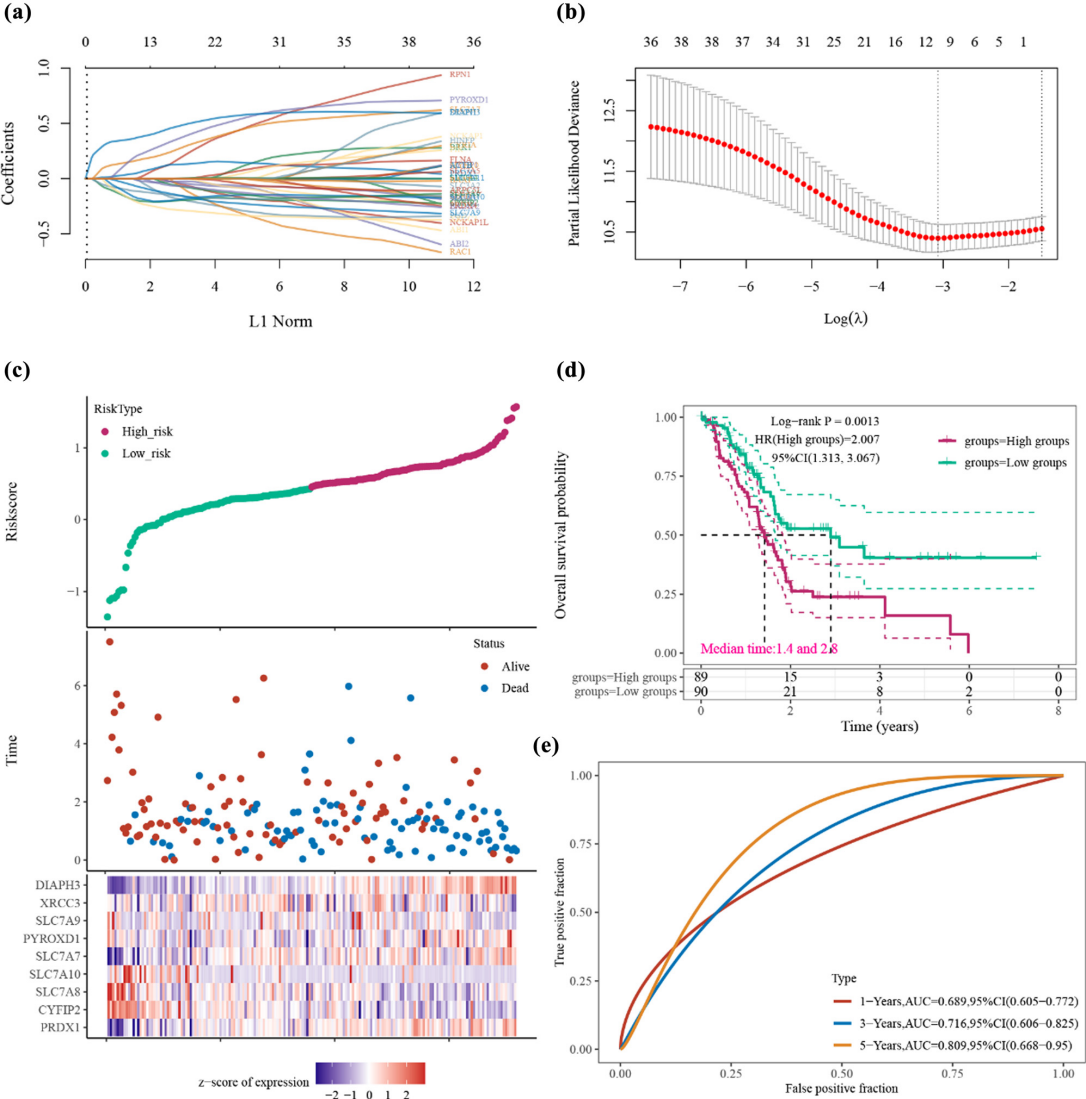
The disulfidptosis signature was evaluated by assessing the performance of a nine-gene signature in the TCGA dataset. (a) Disulfidptosis signatures were constructed using LASSO regression. (b) The number of genes was determined based on the confidence intervals of lambda, identifying the optimal gene set. (c) The risk score, survival time, and expression of the nine-gene signature were analyzed in PAAD. (d) Kaplan–Meier survival analysis compared OS between low- and high-risk score groups in PAAD. (e) ROC curves were generated to assess the predictive performance of the nine-gene signature at 1, 3, and 5 years. OS: overall survival; HR: hazard ratio; ROC: receiver operating characteristic.

### 3.5 Construction of prognosis risk model based on DIGs in TCGA dataset

In order to discover novel prognostic markers for PAAD, we conducted a LASSO regression analysis using data from PAAD patients in the TCGA database. This analysis was based on 40 DRGs. To select the most relevant features, we employed the LASSO regression algorithm with 10-fold cross-validation, as depicted in Figure 6a. Ultimately, we identified nine DRGs that constituted a disulfidptosis signature. The identified genes from the LASSO regression analysis comprised DIAPH3, XRCC3, SLC7A9, PYROXD1, SLC7A7, SLC7A10, SLC7A8, CYFIP2, and PRDX1 (Figure 6b and c). Furthermore, we observed that the overall survival (OS) of patients in the low-risk group was significantly longer compared to the high-risk group (HR = 2.007, 95% CI = 1.313–3.067, *P* = 0.0013), with median survival times of 2.8 years and 1.4 years, respectively (Figure 6d). Additionally, time-dependent ROC curves demonstrated that the accuracy of the nine DRG signatures was equal to or greater than 0.70 for predicting 1-, 3-, and 5-year survival rates (an AUC > 0.7 indicates a high level of accuracy) (Figure 6e).

**Figure 7:**
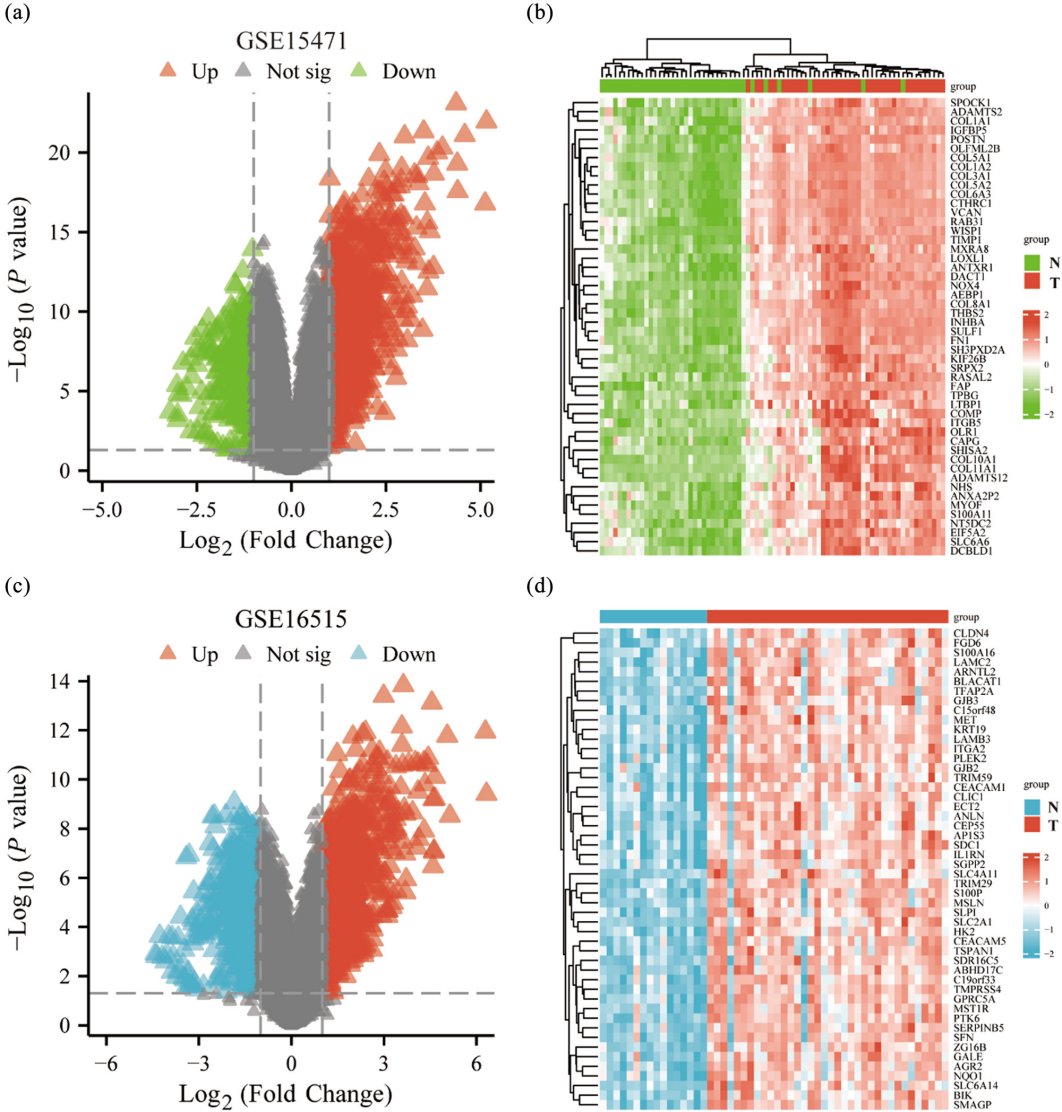
Differential gene analysis to identify differential genes in the GEO dataset. (a) A volcano plot was generated to visualize the differential gene expression in GSE15471 dataset. (b) A heatmap was created to display the differential gene expression patterns in GSE15471 dataset. (c) The differential gene expression in GSE16515 dataset was visualized using a volcano plot. (d) A heatmap was constructed to illustrate the differential gene expression patterns in GSE16515 dataset. N: normal, T: tumor.

### 3.6 Finding differential genes via the GEO datasets

To examine the differential gene expression in PAAD patients, we conducted Limma analysis and generated volcano plots for the datasets GSE15471 (Figure 7a) and GSE16515 (Figure 7c). These volcano plots visually illustrate the differential gene expression patterns in PAAD patients within each dataset. In the dataset GSE15471, a total of 1,315 DEGs were identified, with 1,029 genes up-regulated and 286 genes down-regulated. Similarly, in the dataset GSE16515, a total of 1,512 DEGs were identified, with 959 genes up-regulated and 553 genes down-regulated. Heatmaps showing the top 50 up-regulated genes for GSE15471 (Figure 7b) and GSE16515 (Figure 7d), respectively.

### 3.7 Application of machine learning to the identification of trait genes via the GEO dataset

**Figure 8:**
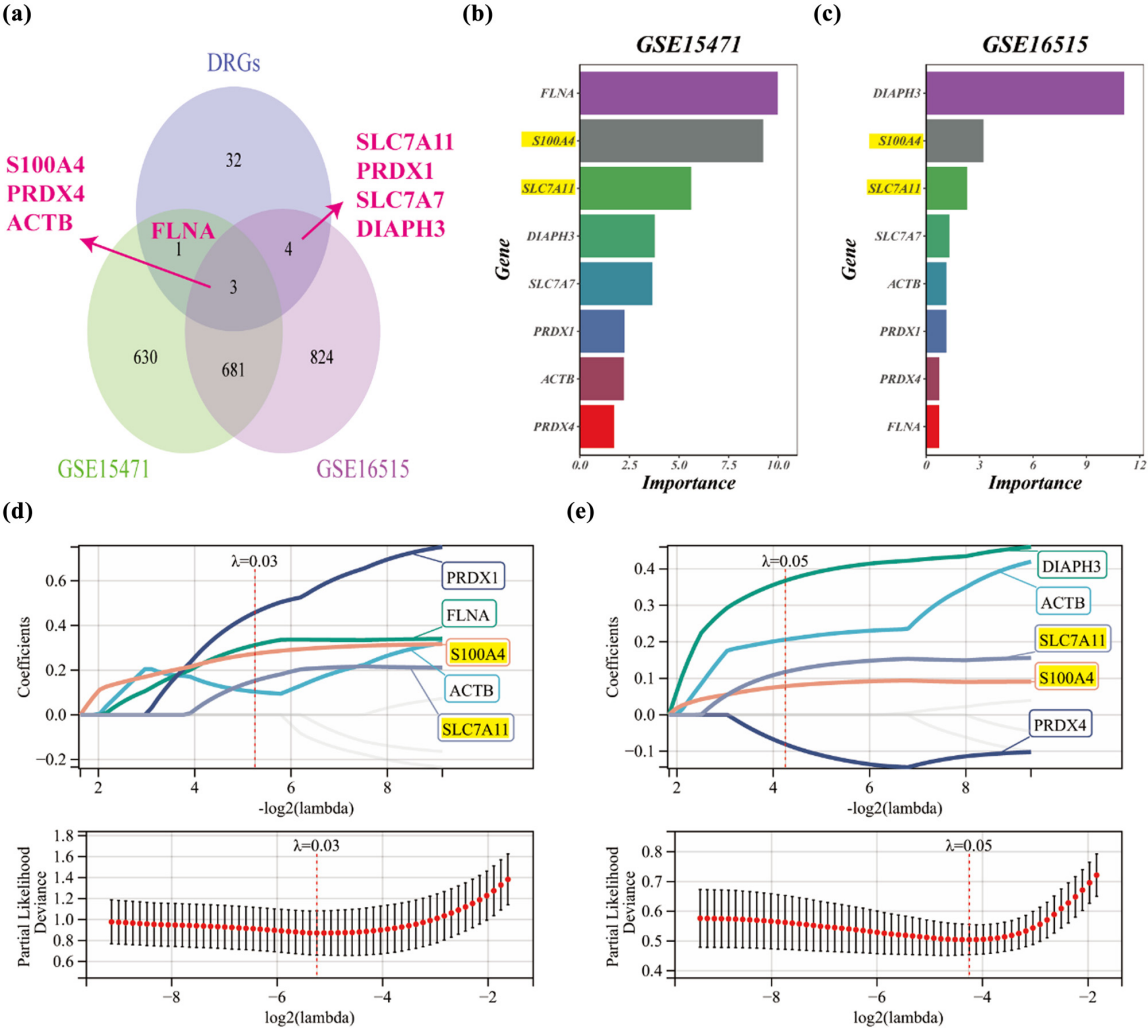
Utilizing machine learning to identify characteristic genes in the GEO dataset. (a) Identification of common trait genes shared by DRGs and differential genes in GSE15471 and GSE16515 datasets. (b) The most relevant DRGs were selected based on GSE15471 dataset using RandomForest analysis. (c) RandomForest analysis was performed to select the most relevant DRGs based on GSE16515 dataset. (d) LASSO regression analysis was employed to select the most relevant trait genes based on GSE15471 dataset. (e) LASSO regression analysis was conducted to select the most relevant trait genes based on GSE16515 dataset.

To explore the overlap between the differential genes and the previously identified DRGs, we employed Venn diagrams known as Wayne plots. These plots depict the intersection between the differential genes obtained from the two datasets. Notably, we found that eight hub genes were common between the differential genes and DRGs, including S100A4, PRDX4, ACTB, FLNA, SLC7A11, PRDX1, SLC7A7, and DIAPH3, as presented in Figure 8a.

Next, we used RandomForest algorithm to screen eight hub genes and construct potential genes based on the GSE15471 (Figure 8b) and GSE16515 (Figure 8c) datasets. We showed the eight central genes in the order of importance and found that genes ACTB, PRDX4, and FLNA exhibited a lack of importance and stability in the PAAD data (Figure 8b and c). In addition, we utilized LASSO regression to identify the most relevant trait genes. By tuning the regularization parameter *λ*, we selected five trait genes. Specifically, when *λ* was set to 0.03 (Figure 8d) and 0.05 (Figure 8e), we identified five trait genes that exhibited strong associations with the analyzed traits. The same result was demonstrated for genes ACTB, PRDX4, and FLNA for lack of contribution and significance in the analysis (Figure 8d and e). Ultimately, S100A4, SLC7A11, PRDX1, SLC7A7, and DIAPH3 was selected as final candidate gene. Among them, genes S100A4 and SLC7A11 have relatively stable importance and stability (Figure 5b–e), and PRDX1, SLC7A7, and DIAPH3 constitute key genes for risk modeling residing in TCGA data (Figure 6c).

### 3.8 Clinical diagnosis and prognostic value analysis of five hub DRGs

**Figure 9:**
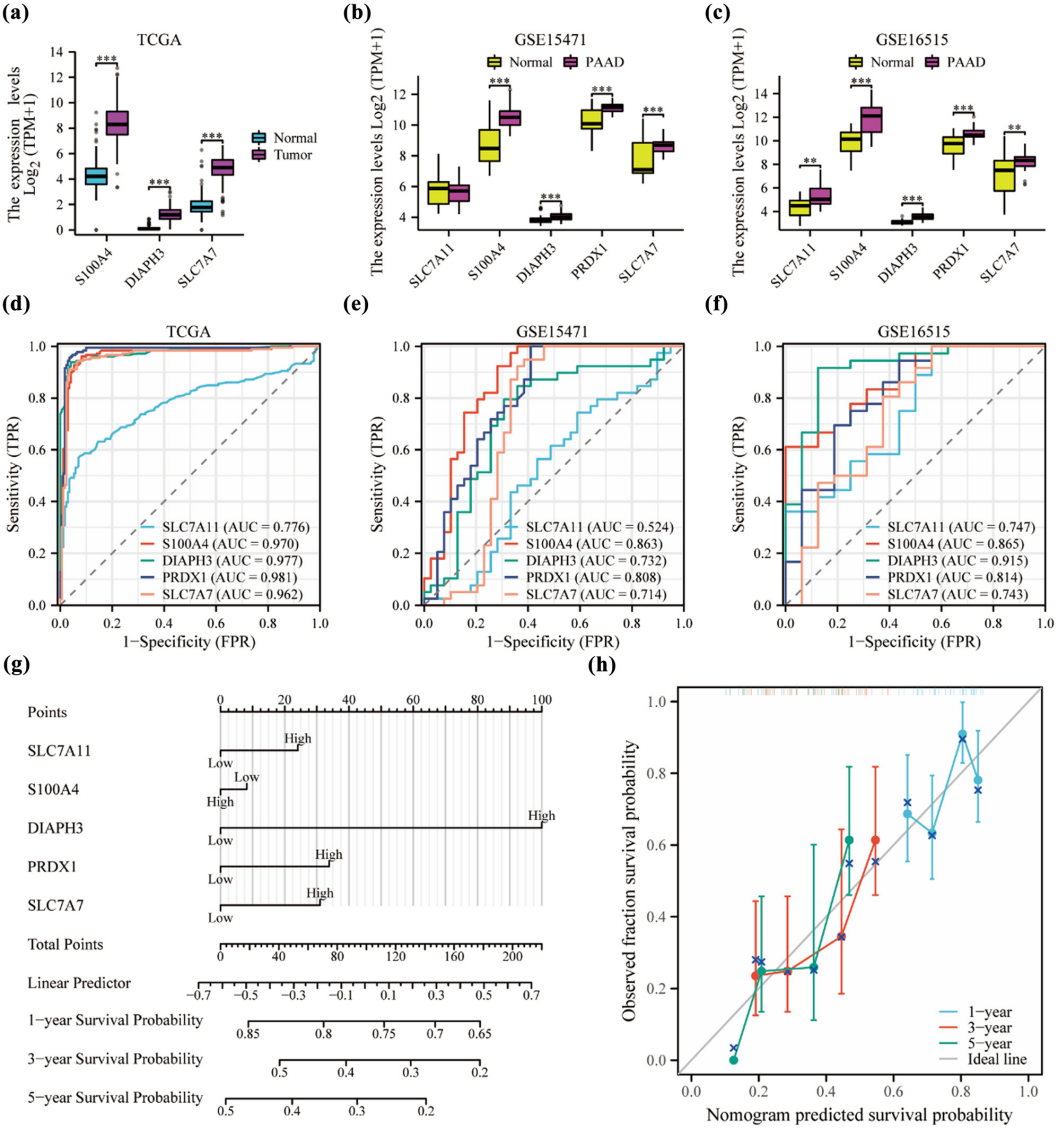
Construction of nomogram for OS prediction based on TCGA. (a) Comparison of the expression distributions of three hub genes between cancer and normal tissues in the TCGA dataset. (b) Comparison of the expression distributions of five hub genes between cancer and normal tissues in the GSE15471 dataset. (c) Comparison of the expression distributions of five hub genes between cancer and normal tissues in the GSE16515 dataset. (d) Evaluation of the performance of five hub genes using ROC curves in the TCGA dataset, (e) GSE15471 dataset, and (f) GSE16515 dataset. (g) Development of a nomogram incorporating five hub genes based on TCGA PAAD data. (h) Calibration curve of the nomogram for predicting OS in TCGA PAAD data at 1, 3, and 5 years. AUC: area under curve; OS: overall survival.

To validate the robustness of our machine learning-based analysis and the significance of the identified key genes, we conducted an analysis using TCGA data. We focused on the five key genes that exhibited significantly high expression in PAAD (all *p* < 0.001) (Figures 1a and 9a). Furthermore, we extended the validation to the independent dataset GSE15471 (Figure 9b) or GSE16515 (all *p* < 0.01) (Figure 9c). These results confirm the reliability and consistency of our findings across different datasets. Furthermore, we analyzed ROC curves for the five trait genes mentioned earlier. In the TCGA dataset, the AUC values for PRDX1, DIAPH3, S100A4, SLC7A7, and SLC7A11 were 0.981, 0.977, 0.970, 0.962, and 0.776, respectively. Similarly, the diagnostic value of key genes is validated in the GEO dataset (Figure 9e and f). These high AUC values indicate the strong predictive power of these genes for distinguishing between different conditions or outcomes in PAAD.

Using the five hub DRG markers, we developed a novel nomogram to estimate the probability of survival at 1, 3, and 5 years for patients diagnosed with PAAD in the TCGA dataset (Figure 9g). This nomogram provides a visual tool for clinicians to assess the likelihood of survival based on the expression levels of the selected DRGs. To validate the predictive accuracy of the nomogram, we conducted calibration curve analysis. The calibration curves, shown in Figure 9h, demonstrate the agreement between the predicted survival probabilities generated by the nomogram and the actual survival outcomes at 1, 3, and 5 years. These results indicate that the nomogram has reliable and accurate predictive capabilities for PAAD survival analysis.

### 3.9 Immune infiltration analysis of five hub DRGs in PAAD

**Figure 10:**
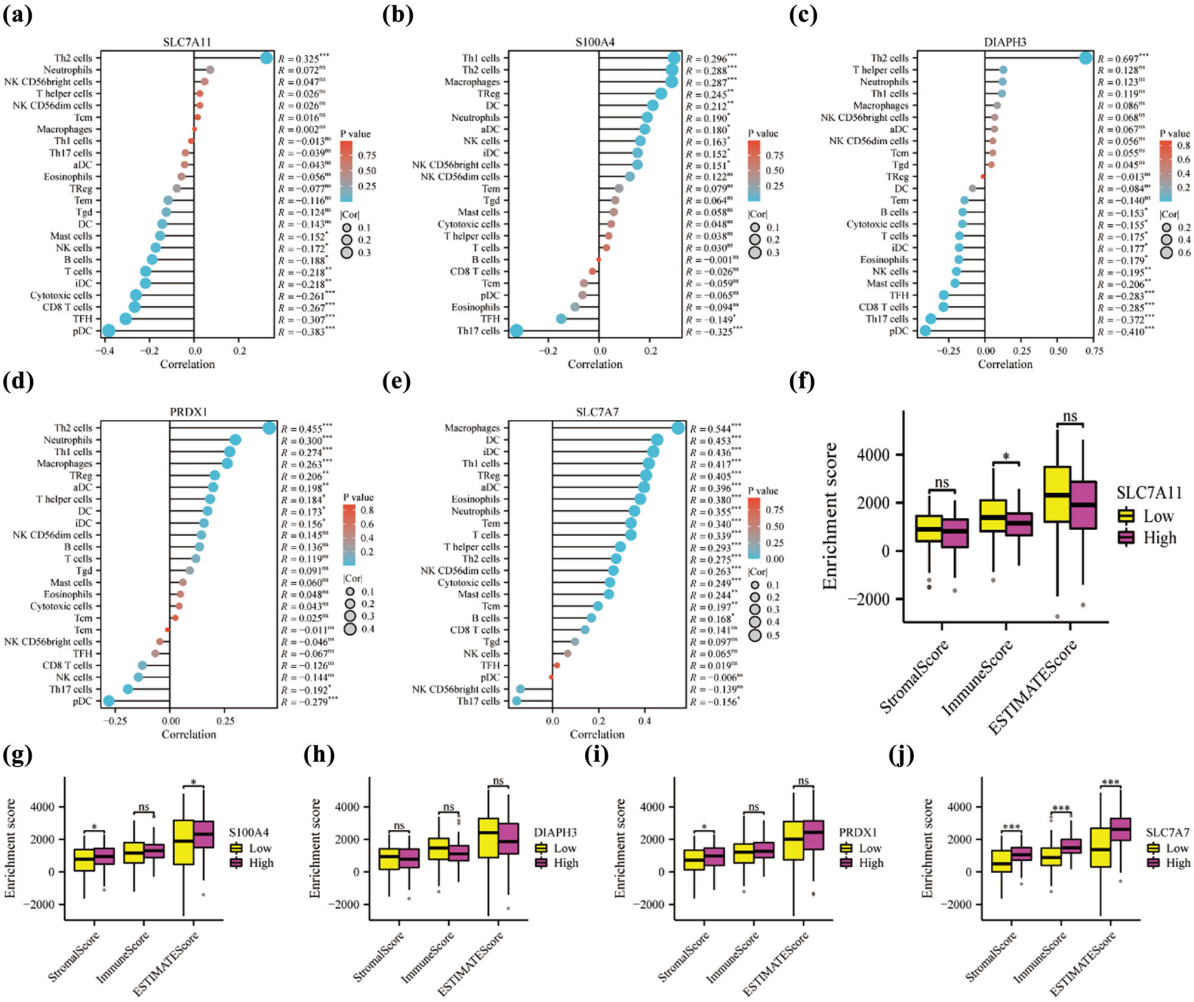
Expression of hub genes in PAAD based on TCGA is correlated with immune infiltration. Correlation between hub gene and multiple immune cells. (a) SLC7A11, (b) S100A4, (c) DIAPH3, (d) PRDX1, and (e) SLC7A7. Hub gene is associated with enrichment score in stromal score, immune score, and ESTIMATE score. (f) SLC7A11, (g) S100A4, (h) DIAPH3, (i) PRDX1, and (j) SLC7A7. np > 0.05, *p < 0.05, **p < 0.01, and ***p < 0.001.

To explore the relationship between the hub genes and immune cell infiltration, we applied the ssGSEA algorithm on 24 immune cell types. The results were analyzed, and the correlation between the hub genes and immune infiltration was visualized using a lollipop plot (Figure 10a–e). Notably, we observed a positive correlation between the hub genes and several immune cell types, including Th2 cells, neutrophils, and macrophages. On the other hand, there was a negative correlation between the hub genes and plasmacytoid dendritic cells (pDC) and Th17 cells. These findings suggest potential interactions and associations between the hub genes and specific immune cell populations involved in the immune response in the context of PAAD.

To investigate the overall level of immune infiltration, we examined three immune infiltration scores that are correlated with the hub genes: StromaScore, ImmuneScore, and ESTIMATEScore. The results revealed significant correlations between certain hub genes and these immune infiltration scores. Specifically, S100A4 (Figure 10g), PRDX1 (Figure 10i), and SLC7A7 (Figure 10j) exhibited a meaningful positive correlation with the immune infiltration scores (all *p* < 0.05). Conversely, SLC7A11 demonstrated a meaningful negative correlation with the ImmuneScore (*p* < 0.05) (Figure 10f). However, DIAPH3 did not show statistical significance in terms of immune infiltration (Figure 10h). These findings suggest that S100A4, PRDX1, SLC7A7, and SLC7A11 may play important roles in modulating the overall immune infiltration in PAAD, while the significance of DIAPH3 in immune infiltration remains uncertain.

## 4 Discussion

PAAD is a highly aggressive tumor with a typically unfavorable prognosis, and the available therapeutic options are limited, underscoring the urgent need for innovative approaches [30]. Consequently, it is essential to assess PAAD prediagnosis and explore novel drugs that target specific functional pathways. In this context, disulfidptosis emerges as a promising avenue for PAAD treatment. Investigating and understanding cell death mechanisms not only enhances our fundamental knowledge of cellular homeostasis but also provides valuable insights for the treatment of various diseases, including cancer. Recent studies have unveiled a novel form of cell death induced by disulfide, termed disulfidptosis [5]. These findings suggest that targeting disulfidptosis through GLUT inhibitors could be an effective strategy for tumor treatment [11]. This study establishes a connection between disulfidptosis and the pathogenesis of PAAD, identifies potential key genes through bioinformatics analysis, and explores potential therapeutic approaches to advance PAAD treatment.

In this study, we conducted a comparative analysis of gene expression between PAAD tumors and normal tissues sourced from the TCGA and GEO databases. The results revealed a significant up-regulation of related genes in tumor tissues compared to normal tissues. To further explore the functional similarities of the identified hub genes, we utilized GeneMANIA to predict genes that shared similar functions, resulting in the identification of 40 similar DRGs. Most of these genes exhibited positive correlations with each other, enhancing the scientific validity and reliability of the gene set. To gain insights into the biological significance of the identified gene set, we performed KEGG and GO enrichment analysis. The analysis revealed enrichment of pathways associated with disulfidptosis and immunity, including regulation of actin cytoskeleton, actin cytoskeleton organization, actin filament-based process, immune response-regulating cell surface receptor signaling pathway involved in phagocytosis and others. These findings provide valuable information about the potential involvement of these pathways in PAAD and highlight their relevance in disease progression and regulation.

At the overall level, we perform a preliminary exploration of DRGs, which are strongly correlated with expression, immune infiltration, mutation, and drug sensitivity in PAAD. We found that differential expression of DRGs and CNV mutations were strongly associated with PAAD prognosis.

Taking S100A4 (myeloid cell leukemia-1) for example, it ranked high in the list of methylation difference and was a significant prognostic risk factor for PAAD. S100A4, also known as metastasin or fibroblast-specific protein-1, is a member of the S100 calcium-binding protein family. It is a small cytoplasmic protein that plays a key role in various cellular processes and has been implicated in cancer progression and metastasis. S100A4 has emerged as a biomarker with predictive value for genetic subtypes, clinical phenotypes, and therapeutic strategies in pancreatic ductal adenocarcinoma [30]. The immune infiltration analysis revealed a positive correlation between PAAD and Tr1 cells, while a negative correlation was observed with neutrophils based on the DRGs. Interestingly, the deletion of these DRGs resulted in increased expression of neutrophils in PAAD. In PAAD, an increase in neutrophils is usually associated with an inflammatory response and alterations in the tumor microenvironment. Studies have shown that in cancer, there is an increased level of neutrophil infiltration in tumor tissue, which may be associated with tumor growth, invasion, and metastasis [31]. The presence of concurrent neutrophils in cancer carries prognostic implications and holds potential as a therapeutic strategy [32]. Studying mutations in DRGs presents an intriguing avenue to explore and better understand the impact of neutrophils in PAAD. This approach can provide valuable insights into the modulation of neutrophil function and its role in the context of PAAD.

There is literature summarizing current knowledge on molecular typing of PAAD and exploring future strategies for using molecular taxonomy to guide treatment development and ultimately conventional therapy with the overall goal of improving this disease [33]. Based on consensus clustering, we identified two PAAD subtypes (C1, C2) by DRGs expression. The subgroups were then further explored for differential genes, and finally found that enrichment analysis of the up-regulated genes in the GO dataset are shown in positive regulation of cell adhesion and T cell activation. On further analysis, C1 subgroup was more significant in relation to immune scores and immune checkpoints, including B cell, T cell CD4+, endothelial cell, and macrophage. This is further evidence of the importance of immunity and disulfidptosis in the development of PAAD.

In this study with the aim to identify new prognostic markers for PAAD, we conducted LASSO regression analysis using 40 DRGs based on PAAD patient data from the TCGA database. Our analysis revealed a disulfidptosis signature consisting of nine genes, namely DIAPH3, XRCC3, SLC7A9, PYROXD1, SLC7A7, SLC7A10, SLC7A8, CYFIP2, and PRDX1. Importantly, we observed that patients in the low-risk group had significantly longer OS compared to those in the high-risk group. Additionally, our time-dependent ROC curves demonstrated an accuracy exceeding 0.70 for predicting 3- and 5-year survival rates.

To validate the reliability of our findings based on TCGA data, we further performed differential gene analysis using the GEO database for PAAD patients. Utilizing the limma analysis, we identified eight DEGs associated with DRGs, including S100A4, PRDX4, ACTB, FLNA, SLC7A11, PRDX1, SLC7A7, and DIAPH3. Subsequently, we employed LASSO regression and the Random Forest algorithm in machine learning to identify the most relevant and stable trait genes, resulting in the selection of S100A4 and SLC7A11. Finally, we identified five hub genes, namely S100A4, SLC7A11, PRDX1, SLC7A7, and DIAPH3, which exhibited high expression levels and demonstrated good diagnostic value in PAAD. Notably, in our prediction model, SLC7A11, DIAPH3, PRDX1, and SLC7A7 showed a causative effect compared to S100A4, with DIAPH3 displaying the highest contribution to the pathogenic effects associated with PAAD carcinogenesis. Overall, this study highlights these five hub genes as potential prognostic markers with significant diagnostic value in PAAD. The findings emphasize the pivotal role of DIAPH3 and provide further insight into the pathogenesis of this disease, which is consistent with previous reports in the literature. The reliability of hub genes as a poor or good prognostic factor ultimately establishes nomogram as an aid to clinicians in the early clinical diagnosis of PAAD.

The progression of tumors is not solely governed by malignant cells but is also influenced by the tumor microenvironment [34]. With ongoing research efforts focused on unraveling the intricacies of the tumor microenvironment, there is immense potential to gain deeper insights into the roles and composition of immune cells within this context, thereby guiding immunotherapy strategies [35]. In our analysis, we investigated the correlation between the five hub genes and immune infiltration to shed light on their potential involvement in the tumor microenvironment. Importantly, we found a positive correlation between the identified hub genes and various immune cell types in our analysis, such as Th2 cells and macrophages. Conversely, we observed a negative correlation between the hub genes and pDC and Th17 cells. Studies have found that BDNF may affect patient prognosis by interacting with tumor-infiltrating Th2 cells, and thus may serve as a potential prognostic biomarker for PAAD [36]. Tumor-associated macrophages play a crucial role as a key component within the tumor microenvironment, orchestrating various processes including angiogenesis, remodeling of the extracellular matrix, cancer cell proliferation, metastasis, immunosuppression, and resistance to chemotherapeutic agents and checkpoint blockade immunotherapy. Macrophages are an essential factor in shaping the tumor microenvironment and influencing tumor progression and treatment outcomes [37]. Research findings have elucidated the critical role of pDCs in antiviral immune responses and their participation in immunopathology, specifically in autoimmune diseases, immunodeficiencies, and cancer [38]. By shedding light on the significance of pDCs in autoimmune diseases, immunodeficiencies, and cancer, these studies have provided valuable insights into the functions of pDCs and their potential therapeutic implications. These findings also underscore the potential involvement of the identified hub genes in modulating immune cell composition and immune responses in PAAD. Therefore, focusing on specific immune cell types in further research may reveal the underlying mechanisms by which these hub genes exert their effects and establish them as promising diagnostic biomarkers for PAAD that are involved in immune regulation. Ultimately, studies investigating hub gene-related DEGs and novel targeted immunotherapies hold the potential to improve the prognosis of PAAD patients and provide healthcare professionals with additional treatment options.

This study has certain limitations that need to be acknowledged. First, the exploration of DRGs in PAAD relied on publicly available databases such as GEO, TCGA, and GTEx, which means that we lacked our own clinical data. As a result, there is a need for human tissue validation to confirm the findings. Furthermore, precise validation through biological experiments is necessary to strengthen the conclusions drawn from this study. To address the challenge of tumor sample heterogeneity, additional techniques such as single-cell analysis or spatial transcriptomics should be employed. These approaches would enable a more comprehensive characterization of the cellular heterogeneity within tumors and provide a deeper understanding of the impact of different cell populations on gene expression patterns and immune infiltration.

## 5 Conclusion

This study has provided important insights into the relationship between DRGs and their expression levels, immune infiltration, mutations, and drug sensitivity in PAAD patients. We have also observed substantial heterogeneity within PAAD patients, both in terms of distinct disulfidptosis subclusters and DEGs. In summary, our findings indicate that S100A4, SLC7A11, PRDX1, SLC7A7, and DIAPH3 may have prognostic significance in PAAD and are associated with immune infiltration levels. These five hub genes hold potential as novel prognostic biomarkers, presenting both opportunities and challenges for the development of new immunotherapeutic strategies. This study represents a scientific and bold endeavor, aiming to push the boundaries of our understanding in the field.
